# PD‐1 inhibitors for non‐small cell lung cancer patients with special issues: Real‐world evidence

**DOI:** 10.1002/cam4.2868

**Published:** 2020-02-06

**Authors:** Seonggyu Byeon, Jang Ho Cho, Hyun Ae Jung, Jong‐Mu Sun, Se‐Hoon Lee, Jin Seok Ahn, Keunchil Park, Myung‐Ju Ahn

**Affiliations:** ^1^ Division of Hematology‐Oncology Department of Medicine Samsung Medical Center Sungkyunkwan University School of Medicine Seoul Korea; ^2^ Division of Hematology‐Oncology Department of Medicine Incheon St. Mary's Hospital Incheon Korea

**Keywords:** autoimmune disease, hepatitis B virus, immune checkpoint inhibitors, interstitial lung disease, non‐small cell lung cancer, tuberculosis

## Abstract

**Background:**

Immune checkpoint inhibitors (ICIs) have provided new therapeutic options for non‐small cell lung cancer(NSCLC) patients. However, due to concerning increases in immune‐related adverse events, clinical trials usually exclude patients with special issues such as viral hepatitis, tuberculosis (Tbc), interstitial lung disease (ILD) and autoimmune disease.

**Methods:**

We retrospectively reviewed the medical records of NSCLC patients who received ICIs, and analyzed the clinical outcomes of patients with special issues.

**Results:**

Between January 2015 and October 2018, 237 patients received ICIs. Of these patients, 26% (61/237) had special issues: 32 had hepatitis B viral (HBV) infections, 20 Tbc, six ILD, one HIV infection, one Behçet's disease and a past HBV infection, and one rheumatoid arthritis. The incidence of hepatitis tended to be higher in patients with HBV infections than in those without (18.8% vs 8.91%, *P* = .082). Severe hepatitis (grade 3 or higher) was more common in HBV‐infected patients (12.5% vs 1.9%, *P* = .0021), but the AEs were well‐managed. During ICI treatment, three of the 20 patients with a history of pulmonary Tbc developed active pulmonary Tbc, considered reactivations. No aggravation of ILD was noted. One RA patient experienced a disease flare and was treated with a low‐dose steroid. There was no significant difference in the overall response rate or progression‐free survival between patients with and without special issues.

**Conclusion:**

Given the relatively low incidence of immune‐related AEs and the comparability of clinical outcomes, ICIs can be treatment option of NSCLC patients with special issues.

## Graphical abstract

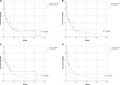

## 1 INTRODUCTION

Immune checkpoint inhibitors (ICIs) have provided new therapeutic options for patients with various cancer types, including NSCLC.^1^, ^2^, ^3^, ^4^, ^5^ In randomized phase III trials on NSCLC, patients treated with nivolumab exhibited better survival than those treated with docetaxel (2‐year OS 23% vs 8% in squamous NSCLC, 29% vs 16% in nonsquamous NSCLC), and the toxicity profile of nivolumab was found to be manageable.^1^ Pembrolizumab also resulted in longer OS (14.9 months vs 8.2 months, *P* = .0002) with a less toxic profile than docetaxel in NSCLC patients.^4^ Pembrolizumab with or without chemotherapy has become the standard first‐line treatment for NSCLC patients without oncogenic drivers.^2^

However, there are theoretical concerns about using ICIs in patients with autoimmune disease or chronic infectious diseases such as chronic hepatitis, pulmonary Tbc, or interstitial lung disease (ILD), as ICIs may dysregulate the host immune balance and cause disease flares by regulating functional T‐cell responses. As a result, patients with such diseases have routinely been excluded from clinical trials.^1^, ^2^, ^4^ In one retrospective study of melanoma patients with autoimmune disease, ipilimumab treatment induced autoimmune disease flares in 27% of patients and severe immune‐related adverse events (irAEs) in 33% of patients.^6^ In another study, anti‐PD‐1 therapy induced disease flares in 38% of melanoma patients with autoimmune disease, and 12% of patients discontinued ICI treatment because of underlying disease flares or irAEs.^7^ Another study investigating anti‐PD‐1 therapy for seven melanoma or NSCLC patients with viral hepatitis revealed that one HCV patient experienced grade 2 ALT elevation and four patients experienced grade 1 ALT elevation.^8^ Regarding ILD, a case series indicated that anti‐PD‐1‐related pneumonitis occurred more frequently in NSCLC patients with ILD than in those without (31% vs 12%, *P* = .014).^9^ In another case report, three lung cancer patients with ILD who were treated with nivolumab did not experience any aggravation of ILD or pneumonitis.^10^ Tuberculosis is still a burdensome disease worldwide. With regard to pulmonary tuberculosis, only seven patients treated with ICIs have been described in previous reports, and the association of ICIs with Tbc reactivation remains ambiguous.^11^, ^12^, ^13^, ^14^, ^15^, ^16^, ^17^

At present, over 10 million people in the United States have an autoimmune disease.^18^ According to a Medicare database analysis, approximately 13.5‐24.6% of lung cancer patients in the United States have an autoimmune disease.^19^ In this context, we analyzed the safety and clinical outcomes of ICIs in NSCLC patients with special issues in real‐world practice.

## 2 PATIENTS AND METHODS

We retrospectively reviewed the medical records of NSCLC patients who received anti‐PD‐1 treatment (pembrolizumab or nivolumab) at Samsung Medical Center from January 2015 to October 2018. We collected medical information including sex; age at diagnosis; pathology; initial stage; laboratory results; response to anti‐PD‐1 treatment; status of HBV infection, HIV infection, tuberculosis, ILD and autoimmune disease; progression‐free survival (PFS); and any toxicity derived from anti‐PD‐1 therapy. The safety profile was set as the primary endpoint variable, and PFS was set as the secondary endpoint variable. Any toxicity was reviewed according to the National Cancer Institute Common Terminology Criteria of Adverse Events (CTCAE), version 4.03. PFS was calculated by the Kaplan‐Meier method from the time of ICI treatment to disease progression or death from any cause. We used Chi‐square and Fisher's exact tests for comparisons of variables. Two‐tailed *P*‐values < .05 were considered significant. All analyses were performed with SPSS ver. 23.0 software (IBM Corporation). This study was approved by the Institutional Review Board of Samsung Medical Center (SMC 2019‐06‐042).

### 2.1 Definitions

A past HBV infection was established if the patient's laboratory findings were negative for hepatitis B surface antigen (HBsAg), positive for hepatitis B virus core IgG antibody (HBcAb), and negative for hepatitis B virus in a DNA test. A chronic HBV infection was noted if the patient's laboratory findings were positive for HBsAg for at least 6 months. ILD was established if the patient's chest computed tomography (CT) findings included ground glass attenuation, reticular opacity, centrilobular consolidation, traction bronchiectasis, or honeycombing. Active pulmonary tuberculosis was noted if the patient's sputum stain and culture were positive for acid‐fast bacillus. Old Tbc was established if the patient had chest CT findings compatible with fibronodular or calcified lesions with or without fibrotic scars or medical history of pulmonary tuberculosis diagnosis with or without antituberculosis treatment.

## 3 RESULTS

From January 2015 to October 2018, 237 NSCLC patients were treated with PD‐1 inhibitors. Among them, 24.4% (58/237) had special issues: 32 patients had HBV infections (16 past HBV infections and 16 chronic HBV infections), 20 had a history of pulmonary Tbc, six had ILD, one had HIV and two had autoimmune diseases (Behçet's disease and rheumatoid arthritis [RA], respectively). Two patients had past HBV infections and old pulmonary Tbc, and one patient had a past HBV infection and Behçet's disease.

The median age at diagnosis was 60 years (range 35‐86). There were 167 male and 70 female patients. The most common type of NSCLC was adenocarcinoma (64.5%), followed by squamous cell carcinoma (25.3%). Most patients (64%) were diagnosed with stage IV NSCLC during their initial work‐up. Bone was the most common metastatic site (38%), followed by the brain (26.6%) and liver (21.5%). In total, 122 patients received anti‐PD‐1 treatment as the third or fourth line of chemotherapy, 90 patients received it as the first or second line, and 25 patients received it as the sixth or greater line. Nivolumab was administered to 147 patients, while pembrolizumab was administered to 90 patients. The patients’ clinicopathologic characteristics are summarized in Table 1.

**Table 1:** Baseline Characteristics of the Total Population

Baseline Characteristics	N = 237
Age, years, median (range)	60 (35‐86)
Sex	
Male	167
Female	70
Pathology	
ADC	154
SqCC	60
Others	23
Smoking	
Current	81
Ex	73
Never	83
ECOG performance	
0	1
1	174
2	62
Stage at diagnosis*	
I‐III	85
IV	152
Metastasis	
Brain	63
Liver	51
Bone	90
Anti‐PD1 line	
I‐II	90
III‐V	122
VI or more	25
Anti‐PD1 treatment	
Nivolumab	147
Pembrolizumab	90

Abbreviations: ADC, adenocarcinoma; ECOG, Eastern Cooperative Oncology Group; PD1, programmed cell death 1; SqCC, squamous cell carcinoma.

* Union for International Cancer Control (UICC) TNM classification and clinical staging system.

### 3.1 Adverse events

There were 149 treatment‐related adverse events in the total population. Hepatitis was the most common adverse event (10.13%), followed by fatigue and anorexia pruritus (8.86% each). In terms of severe AEs (grade 3 or higher), hepatitis was the most common (3.38%), followed by pneumonitis (1.69%) and myalgia (0.84%). The toxicity profile of the total population is summarized in Table 2.

**Table 2:** Treatment‐Related Adverse Events

Adverse events	All Grades	%	Grade ≥ 3	%
Hepatitis	25	10.55	9	3.80
Jaundice	11	4.64	1	0.42
Pneumonitis	9	3.80	4	1.69
Fatigue	21	8.86	1	0.42
Anorexia	21	8.86	0	0.00
Nausea/vomiting	7	2.95	0	0.00
Constipation	2	0.84	0	0.00
Diarrhea	4	1.69	1	0.42
Pruritus	21	8.86	0	0.00
Skin rash	14	5.91	0	0.00
Mucositis	3	1.27	0	0.00
Nail change	1	0.42	0	0.00
Myalgia	6	2.53	2	0.84
Neuropathy	2	0.84	0	0.00
Neutropenia	1	0.42	1	0.42
Lymphopenia	1	0.42	0	0.00
Cough	1	0.42	0	0.00
Hyperpigmentation	1	0.42	0	0.00
Hypothyroidism	2	0.42		
Hyperglycemia	1	0.42		
Adrenal insufficiency	1	0.42		

Eleven patients discontinued their anti‐PD‐1 treatment due to adverse events: one patient discontinued due to grade 4 hepatitis, four patients due to grade 3 pneumonitis, one due to grade 2 anorexia, one due to grade 3 myalgia, two due to grade 3 fatigue, one due to grade 4 neutropenia, and one due to demyelinating polyneuropathy and adrenal insufficiency.

Of note, two patients without underlying ILD newly developed grade 4 immune‐related pneumonitis during nivolumab treatment. Their CT scans exhibited organizing pneumonia patterns compatible with ICI‐induced pneumonitis. Steroid therapy resolved these cases of immune‐related pneumonitis, but these two patients had sequelae of lung fibrosis.

### 3.2 HBV population

Among the 32 HBV‐infected patients, six patients experienced hepatitis. Hepatitis occurred more frequently in HBV‐infected patients than in non‐HBV patients, but the difference was not statistically significant (18.8% vs 8.91%, *P* = .082). In contrast, the incidence of severe hepatitis (grade 3 or higher) was significantly higher in HBV‐infected patients than in non‐HBV patients (12.5% vs 1.9%, *P* = .0021). The HBV infection status (past vs chronic) was not associated with hepatitis (*P* = .654).

Fourteen patients received anti‐HBV therapy (10 with entecavir, one with tenofovir, and three with lamivudine) prior to anti‐PD‐1 treatment. Interestingly, anti‐HBV therapy prior to or during anti‐PD‐1 treatment was not associated with the incidence of hepatitis events (*P* = .608).

Among the 16 chronic hepatitis patients, three patients experienced viral reactivations or flares. One patient experienced HBV DNA seroconversion from undetectable to 1484 IU/mL after 1 month of pembrolizumab treatment. The HBV DNA returned to an undetectable level after 1 month of entecavir, but the patient did not restart the pembrolizumab treatment due to cancer progression. In another patient, the HBV DNA level increased from 70 IU/mL to 2813 IU/mL after one cycle of nivolumab. The patient had been taking tenofovir prior to starting nivolumab, but died due to NSCLC progression 1 month later. Another patient had an HBV DNA level of 1553 IU/mL while taking entecavir before pembrolizumab treatment. His HBV DNA level rose to 11 317 IU/mL after 1 month of pembrolizumab treatment, but spontaneously dropped to 599 IU/mL despite his continuation of pembrolizumab and entecavir treatment.

Among the HBV‐infected patients, two patients discontinued their anti‐PD‐1 therapy due to AEs. One patient experienced grade 4 hepatitis, hypothyroidism, and hyperglycemia, while the other patient experienced grade 2 pneumonitis. The characteristics, anti‐PD‐1 treatment types, HBV DNA test results, AST/ALT elevations, and irAEs of all the HBV patients are summarized in Table 3.

**Table 3:** Safety and treatment outcomes of anti‐PD‐1 treatment for the HBV population

Pt. number	HBV status	Sex	Age at treatment	Histology	Anti‐PD1	Viral load prior to and post‐ICI	Anti‐HBV tx.	Best response	CTCAE AST/ALT elevation	irAE	Comment
1	Past HBV	M	73	ADC	Nivo	pre: Undetectable post: Undetectable	Lamivudine prior to anti‐PD1	SD	AST G3 ALT G4	Hyperglycemia G3 Hypothyroidism G2	
2	Past HBV	M	57	SqCC	Nivo	Prior: Undetectable Post: N/A		not evaluable	AST G1 ALT G1		Liver metastasis progression F/u loss
3	Past HBV	M	68	ADC	Nivo	pre: Undetectable post: Undetectable		PD			
4	Past HBV	M	66	ADC	Nivo	pre: Undetectable post: Undetectable		SD	AST G3 ALT G4	Pneumonitis G4	
5	Past HBV	M	58	ADC	Pembro	pre: Undetectable post: Undetectable		PR		Pneumonitis G2	
6	Past HBV	M	62	SqCC	Pembro	N/A		PR			
7	Past HBV	F	63	ADC	Nivo	pre: Undetectable post: Undetectable		PR			
8	Past HBV	F	61	ADC	Pembro	pre: Undetectable post: Undetectable		PD	AST G3 ALT G2		
9	Past HBV	F	64	ADC	Nivo	pre: Undetectable post: Undetectable		SD	AST G1 ALT G1		
10	Past HBV	M	65	ADC	Nivo	pre: Undetectable post: Undetectable		PD	AST G1 ALT G1		Liver metastasis progression
11	Past HBV	M	61	SqCC	Pembro	N/A		PD			
12	Past HBV	M	68	ADC	Nivo	Prior: Undetectable Post: N/A		PD			
13	Past HBV	F	61	ADC	Pembro	Prior: Undetectable Post: N/A		SD	AST G3 ALT G3		D/t pancreatitis
14	Past HBV	M	57	SqCC	Nivo	Prior: Undetectable Post: N/A		PD			
15	Past HBV	M	76	ADC	Pembro	Prior: Undetectable Post: N/A		PR			
16	Past HBV	M	64	ADC	Pembro	N/A		SD			
17	Chronic HBV	M	66	ADC	Nivo	Prior: Undetectable Post: N/A	Entecavir prior to anti‐PD1	not evaluable			F/u loss
18	Chronic HBV	M	61	SqCC	Nivo	pre: Undetectable post: Undetectable	Entecavir prior to anti‐PD1	SD	AST G2 ALT G3	Fatigue G2	
19	Chronic HBV	M	88	SqCC	Nivo	pre: Undetectable post: Undetectable	Lamivudine prior to anti‐PD1	SD			
20	Chronic HBV	M	59	P/D carcinoma	Nivo	Prior: 70 Post: 2813	Tenofovir prior to anti‐PD1	not evaluable	AST G1		Dz. progression Expired
21	Chronic HBV	M	58	SqCC	Nivo	N/A	Lamivudine prior to anti‐PD1	PD			HBsAg (+) ‐> HBsAg (−)
22	Chronic HBV	M	79	SqCC	Nivo	pre: Undetectable post: Undetectable		PD			
23	Chronic HBV	F	62	ADC	Pembro	Prior: 2081 Post: N/A	Entecavir prior to anti‐PD1	not evaluable			F/u loss
24	Chronic HBV	M	60	SqCC	Pembro	Prior: Undetectable Post: N/A	Entecavir prior to anti‐PD1	not evaluable	AST G3 ALT G3		Dz. progression Expired
25	Chronic HBV	F	54	ADC	Pembro	pre: Undetectable post: Undetectable	Entecavir prior to anti‐PD1	SD			
26	Chronic HBV	M	69	ADC	Pembro	Prior: Undetectable Post: N/A	Entecavir prior to anti‐PD1	PD			Dz. progression Expired
27	Chronic HBV	F	40	ADC	Pembro	Prior: 814 405 Post: N/A	Entecavir prior to anti‐PD1	SD			
28	Chronic HBV	M	73	ADC	Pembro	Prior: 326 Post: N/A		not evaluable	AST G3 ALT G1		Expired d/t sepsis
29	Chronic HBV	M	60	ADC	Pembro	Prior: Undetectable Post: 1484 ‐>undetectable	Entecavir prior to anti‐PD1	PD		DNA seroconversion	
30	Chronic HBV	M	59	ADC	Pembro	Prior: Undetectable Post: N/A	Entecavir prior to anti‐PD1	PD			
31	Chronic HBV	M	75	ADC	Pembro	N/A	Lamivudine prior to anti‐PD1	PR			
32	Chronic HBV	M	45	Adenosquamous	Pembro	Prior: 1553 Post: 11 317‐>599	Entecavir prior to anti‐PD1	PR			

Abbreviations: ADC, adenocarcinoma; ALT, alanine aminotransferase; AST, aspartate aminotransferase; DNA, deoxyribonucleic acid; HBV, Hepatitis B virus; irAE, immune‐related adverse event; PD, progressive disease; PR, partial response; SqCC, squamous cell carcinoma; SD, stable disease.

### 3.3 Interstitial lung disease population

Six patients had underlying ILD before anti‐PD‐1 treatment. None of these patients experienced any aggravation of ILD or developed immune‐related pneumonitis. The ILD patterns and treatment outcomes of these patients are summarized in Table 4.

**Table 4:** Characteristics and anti‐PD‐1 treatment outcomes of the ILD population

Pt. number	Sex	Age at treatment	Histology	Anti‐PD1	ILD pattern	Best response	irAE	Comment
1	M	63	Unknown	Nivo	UIP	PD		
2	M	63	ADC	Nivo	NSIP	PR		
3	M	67	SqCC	Pembro	UIP	SD		
4	M	63	SqCC	Pembro	UIP	not evaluable		Expired d/t PD
5	M	72	Adenosquamous	Pembro	UIP	not evaluable		F/u loss
6	F	59	ADC	Pembro	UIP	PD		

Abbreviations: ADC, adenocarcinoma; COP, cryptogenic organizing pneumonia; ILD, interstitial lung disease; Nivo, nivolumab; NSIP, nonspecific interstitial pneumonia; PD, progressive disease; Pembro, pembrolizumab; PR, partial response; SD, stable disease; SqCC, squamous cell carcinoma; UIP, usual interstitial pneumonia.

### 3.4 Pulmonary tuberculosis population

Among the 20 patients with a history of pulmonary tuberculosis, three patients developed active pulmonary Tbc during or after their anti‐PD‐1 treatment. One patient had received 43 cycles of nivolumab therapy and achieved a partial response by the RECIST criteria. However, sustained pneumonic consolidation with a cavitary lesion in the right upper lung was noted after the 22nd cycle of nivolumab therapy. A bronchoalveolar lavage and an acid‐fast bacillus culture of bronchoalveolar lavage fluid revealed a mycobacterium tuberculosis complex with trace stain results. Thus, the patient took anti‐Tbc medication for 6 months while continuing nivolumab therapy without interruption. Another patient developed pulmonary Tbc after 1 month of pembrolizumab treatment, and thus started anti‐Tbc medication while continuing pembrolizumab therapy. After 3 months, cancer progression was documented. The patient continued taking the anti‐Tbc medication for a total of 6 months and then refused further treatment. The last patient received nivolumab treatment for 2 months, and discontinued because of disease progression. After 4 months, she developed pulmonary Tbc. She requested a referral to a nearby hospital and was lost to follow‐up.

The remaining 17 patients with old pulmonary Tbc did not experience reactivation of pulmonary Tbc. Also, none of the patients developed extrapulmonary Tbc disease. The characteristics and anti‐PD‐1 treatment outcomes of the Tbc population are summarized in Table 5.

**Table 5:** Characteristics and anti‐PD‐1 treatment outcomes of the Tbc population

Pt. number	Sex	Age at treatment	Histology	Anti‐PD1	TBc	TBc diagnosis	Best response	irAE	Comment
1	M	57	SqCC	Nivo	old TBc		Not evaluable		F/u loss
2	M	68	ADC	Nivo	pul. TBc s/p Tx.	Prior to anti‐PD1	PD		
3	M	58	Poorly diff. carcinoma	Nivo	old TBc		PD		
4	F	46	SqCC	Nivo	pul. TBc s/p Tx.	Prior to anti‐PD1	not evaluable		F/u loss
5	F	57	ADC	Nivo	pul. TBc	After anti‐PD1	PD		Tb reactivation F/u loss
6	F	65	ADC	Nivo	old TBc		SD	Pneumonitis G3	
7	M	75	SqCC	Nivo	old TBc		PD		
8	M	51	ADC	Nivo	old TBc		PR	Hepatitis G1	
9	M	51	ADC	Nivo	old TBc		SD		
10	F	56	ADC	Nivo	pul. TBc s/p Tx.	Prior to anti‐PD1	PD		
11	M	69	SqCC	Nivo	pul. TBc s/p Tx.	Prior to anti‐PD1	PD		
12	M	61	ADC	Nivo	pul. TBc s/p Tx.	During anti‐PD1	PR	Hypothyroidism Hepatitis G1	Tb reactivation cured
13	M	57	ADC	Nivo	old TBc		PD		
14	M	54	Pleomorphic carcinoma	Nivo	old TBc		SD		
15	M	85	ADC	Nivo	old TBc		PR		
16	M	64	ADC	Nivo	old TBc		not evaluable		Expired d/t PD
17	M	71	Adenosquamous	Pembro	old TBc		PR		
18	M	66	ADC	Pembro	old TBc		PD		
19	M	55	ADC	Pembro	pul. TBc s/p Tx.	Prior to anti‐PD1	SD		
20	F	84	SqCC	Pembro	pul. TBc s/p Tx.	During anti‐PD1	PD		Tb reactivation cured

Abbreviations: ADC, adenocarcinoma; Nivo, nivolumab; Pembro, pembrolizumab; PD, progressive disease; PD1, programmed cell death 1; PR, partial response; SD, stable disease; SqCC, squamous cell carcinoma; TBc, tuberculosis.

### 3.5 HIV patient

One HIV‐infected NSCLC patient received nivolumab as a second‐line therapy for NSCLC after starting antiretroviral therapy. He stopped receiving nivolumab treatment after 1 month due to disease progression, and died after 2 months due to disease progression despite further chemotherapy. During nivolumab treatment, he did not experience any adverse events.

### 3.6 Autoimmune disease

One patient had a past HBV infection and Behçet's disease. He received nivolumab treatment and did not experience any HBV event or aggravation of Behçet's disease. Another patient had seropositive RA and received 12 cycles of nivolumab treatment. After the first cycle of nivolumab, he experienced grade 2 arthralgia and was administered 5 mg of prednisolone plus 400 mg of hydroxychloroquine daily by a rheumatologist. He continued receiving nivolumab during the steroid and hydroxychloroquine treatment. After 3 months, the steroid was tapered off, and the patient continued receiving nivolumab for the next 8 months until cancer progression.

### 3.7 Overall response rate and progression‐free survival

**Figure 1:**
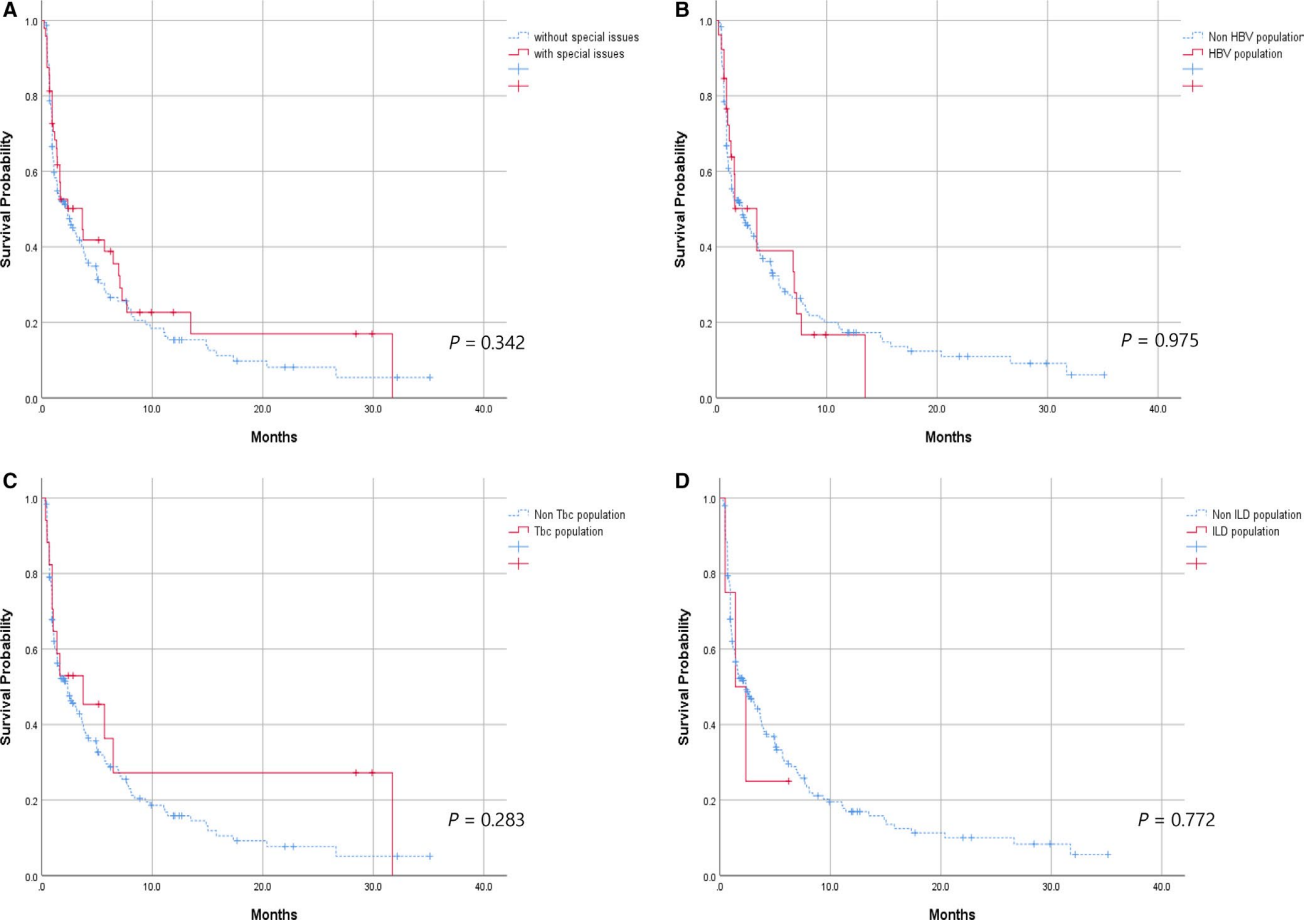
Kaplan‐Meier curves. A, PFS of eligible NSCLC patients with or without special issues. B, PFS of eligible NSCLC patients with or without HBV infections. C, PFS of eligible NSCLC patients with or without tuberculosis. D, PFS of eligible NSCLC patients with or without ILD

Among the 237 patients, 199 were eligible for response evaluation by the RECIST criteria. The overall response rate (ORR) for the eligible population was 25.6% (51/199). The ORR did not differ between NSCLC patients with and without special issues (26% vs 25.5%, respectively). The ORR was 23.1% (6/26) for the HBV population, 23.5% (4/17) for the Tbc population, and 50% (3/6) for the ILD population. The median PFS time for the eligible population was 2.5 months (95% CI 1.30‐3.82). The PFS time did not differ significantly between NSCLC patients with and without special issues (3.6 months vs 2.3 months, *P* = .342). There were also no statistically significant differences in PFS between the HBV and non‐HBV populations (3.6 months vs 2.3 months, *P* = .975), between the Tbc and non‐Tbc populations (3.7 months vs 2.3 months, *P* = .283), or between the ILD and non‐ILD populations (1.4 months vs 2.3 months, *P* = .772) (Figure 1).

## 4 DISCUSSION

In our study, 149 treatment‐related adverse events (62.4%) were observed, consistent with the rates in previous studies (68% in the Checkmate 017/057 trial, 73% in the KEYNOTE‐024 trial).^1^, ^2^ The AEs were mostly low‐grade (grade 1 or 2, 87.9%) and manageable. Eleven patients (4.6%) discontinued their anti‐PD‐1 treatment due to AEs; this rate of discontinuation was comparable to that in a previous study.^1^

It is noteworthy that aminotransferase elevation was the most frequent AE among NSCLC patients in our study. The frequency of this AE (10.5%) was higher than those in previous studies; the incidence of autoimmune hepatotoxicity was reported to be 3‐9% with CTLA‐4 inhibitors^20^ and 3‐4% with PD‐1 inhibitors.^3^ In a phase 1/2 trial of nivolumab treatment in HCC patients with or without HBV or HCV infections, 26% of HCV‐infected patients (13/50) and 8% of HBV‐infected patients (4/51) exhibited AST or ALT elevation.^21^ Therefore, the higher incidence of liver enzyme elevation in the present study could be attributed to the higher proportion of patients with HBV infections (13.5%, 32/237).

In our study, among the 25 NSCLC patients with any grade of AST/ALT elevation, seven were HBV patients (7/32 patients, 21.8%) and 18 were non‐HBV patients (18/205 patients, 8.8%). In particular, four of 16 patients (25%) with past HBV infections and three of 16 patients (18%) with chronic HBV infections developed hepatitis during or after anti‐PD‐1 therapy. In the case of severe AST/ALT elevation (grade 3 or higher), five of nine patients were HBV patients. Although the combined incidence of all grades of hepatitis did not differ significantly between NSCLC patients with and without HBV infections, severe hepatitis was more common in patients with HBV infections (*P* = .011, Linear by Linear).

With regard to hepatitis B viral reactivations or flares, there was no evidence of these events in HBV‐infected HCC patients treated with nivolumab in a previous study.^21^ However, in that study, all the HBV patients received effective antiviral therapy, which could have suppressed HBV reactivations or flares.^21^ In our study, three NSCLC patients with HBV infections developed viral reactivations or flares. Two patients experienced HBV DNA increases after one cycle of immunotherapy (nivolumab and pembrolizumab, respectively), despite receiving anti‐HBV therapy prior to the immunotherapy. Another patient experienced HBV seroconversion after one cycle of pembrolizumab treatment, despite receiving entecavir prior to the immunotherapy. Our findings suggest that regular hepatitis viral status checkups should be required when ICI treatments are applied to patients with hepatitis infections. So far, there have been no reports comparing the incidence of hepatitis between hepatitis virus‐infected and noninfected patients treated with ICIs. A prospective study will be needed to establish the true safety of ICI treatment in patients with viral hepatitis, especially in Asian countries with a high prevalence of hepatitis.

To our knowledge, there have only been seven reports about acute Tbc infections or reactivations in patients receiving ICI treatment.^11^, ^12^, ^13^, ^14^, ^15^, ^16^ In this study, Three patients were newly diagnosed with pulmonary Tbc during their anti‐PD‐1 treatment. Considering the high prevalence of tuberculosis in Korea^22^ and the age of the patients, these three cases were considered to be Tbc reactivations rather than acute tuberculosis infections. Two hypotheses regarding Tbc activation during ICI treatment were suggested by Reungwetwattana et al^17^ The first was that a host response similar to immune reconstitution inflammatory syndrome occurs, while the second was that Tbc activation is due to ICI‐induced lymphopenia.^17^ A recent study indicated that PD‐1 and PD‐L1 inhibitory receptors are overexpressed on the mononuclear cells of tuberculosis patients, suggesting that mycobacteria can exploit PD‐1/PD‐L1 pathways to evade the host response.^23^ Of interest, one of the two reactivated Tbc patients in our cohort developed lymphopenia after pembrolizumab treatment. We do not know whether the lymphopenia truly reactivated the Tbc in this case; nevertheless, these findings imply that in areas like Korea with a high prevalence of Tbc, a Tbc screening test such as the interferon‐gamma release assay (IGRA) should be performed to determine whether a patient has latent Tbc before ICI treatment is started.

ILD is present in about 15% of patients at their initial diagnosis of lung cancer,^24^, ^25^ and is considered to limit the effectiveness of lung cancer treatment.^26^, ^27^ Conventional platinum‐based chemotherapy can acutely exacerbate ILD.^27^ Moreover, EGFR tyrosine kinase inhibitors, a novel standard therapy for EGFR‐mutant NSCLC patients, were found to cause pneumonitis more commonly in NSCLC patients with preexisting ILD than in those without.^28^ On a theoretical basis, ICIs have the potential to worsen ILD in NSCLC patients, so most clinical trials have excluded patients with preexisting ILD.^1^, ^2^, ^3^, ^4^, ^5^, ^9^, ^29^, ^30^ In general, ICI‐associated pneumonitis occurs in 3‐12% of NSCLC patients.^2^, ^31^, ^32^, ^33^, ^34^ Only a few retrospective studies reporting the clinical use of ICIs in NSCLC patients with ILD are available. One retrospective study indicated that ICI‐associated pneumonitis was more common in ILD NSCLC patients than in non‐ILD NSCLC patients (31% vs 12%).^9^ In another study, pneumonitis was observed more frequently in patients with preexisting ILD than in those without, but the difference was not statistically significant (29% vs 11%, *P* = .08).^30^ In our study, none of the patients with preexisting ILD experienced ILD‐AEs or pneumonitis, although the number of the patients was small (n = 6). PFS did not differ significantly between patients with and without ILD (not reached vs 2.56 months, *P* = .248), consistent with previous studies.^9^, ^29^, ^30^

In our study, two NSCLC patients with autoimmune disease received anti‐PD‐1 treatment. The Behçet's disease patient did not experience a flare or exacerbation of the underlying autoimmune disease. However, the NSCLC patient with RA experienced a flare of RA requiring steroid treatment. One retrospective study indicated that eight of 30 melanoma patients with autoimmune disease (27%) experienced an autoimmune disease exacerbation necessitating steroid treatment after using ipilimumab, including five of six RA patients.^6^ In another study, 20 of 52 melanoma patients (38%) experienced autoimmune disease flares requiring immunosuppression after anti‐PD‐1 treatment (nivolumab or pembrolizumab), including seven of 13 RA patients.^7^ According to a systematic review, 15 of 20 RA patients (75%) treated with ICIs developed AEs, including seven (35%) who had RA flares and five (25%) who had de novo irAEs. All of these patients required immunosuppressive treatment.^35^ Taken together, these results indicate that caution should be taken when ICIs are administered to patients with autoimmune disease, and close follow‐up is essential.

Our study had several limitations. Given the retrospective study design and the relatively small number of patients in each special issue category, the included patients might not be representative of NSCLC patients with HBV infections, Tbc, or ILD. Further, there may have been a selection bias for using ICIs in these patients. Additionally, since the disease evaluation and treatment depended on the physician's discretion rather than a protocol, there may have been a detection bias. Nevertheless, our study has provided clinically meaningful real‐world data from clinical practice.

Our study suggests that ICIs can be used in patients having so‐called “ineligible diseases” for clinical trials, such as viral hepatitis, tuberculosis, ILD, and autoimmune disease. The AEs of ICIs were generally manageable, and the treatment outcomes were comparable in NSCLC patients with and without these diseases. A recent study on prophylactic tumor necrosis factor alpha (TNF‐a) neutralization in combination with anti‐PD‐1 and anti‐CTLA‐4 immunotherapy suggested that TNF blockade therapy can ameliorate immune‐related toxicity while maintaining the antitumor efficacy of immunotherapy.^36^ It remains to be determined whether the same strategy can be applied to patients with chronic infections or autoimmune disease. Further prospective studies will be needed to determine the safety of ICIs in NSCLC patients with special issues.

## AUTHOR CONTRIBUTIONS

SB and MA designed the study and drafted the manuscript. JHC and HAJ contributed the materials. SL and JSA interpreted the data. KP conceived the study. All the authors read and approved the final manuscript.

## Data Availability

The data will be provided upon the request.
